# Clinical Outcomes in Placebo-Injection and Injection-Free Cohorts in Birch Pollen Allergy: An Exploratory Cross-Study Comparison

**DOI:** 10.3390/jcm15197637

**Published:** 2026-10-02

**Authors:** Esther Raskopf, Carmen Fierro Ballester, Hacer Sahin, Nadine Katzke, Christian Neuhof, Laura Day, Anna Rybachuk, Silke Allekotte, Miguel Casanovas, José Luis Subiza, Sandra del Pozo

**Affiliations:** 1 ClinCompetence Cologne GmbH, 50933 Cologne, Germany; 2 Institute of Medical Statistics and Computational Biology, Faculty of Medicine, University of Cologne, 50931 Cologne, Germany; 3 Inmunotek S.L., 28805 Alcalá de Henares, Madrid, Spain

**Keywords:** allergen immunotherapy, placebo effect, birch pollen allergy, seasonal allergic rhinitis, placebo-controlled trial, patient-reported outcomes, combined symptom and medication score, therapeutic context

## Abstract

**Background/Objectives:** Placebo responses can complicate the interpretation of allergen immunotherapy (AIT) trials because symptom- and medication-based outcomes may be influenced by the trial context in addition to treatment-specific effects. Injection-free comparison cohorts are rarely available in conventional double-blind, placebo-controlled AIT trials. We therefore conducted an exploratory post hoc cross-study comparison of clinical outcomes in two placebo-injection cohorts and an injection-free cohort of birch pollen-allergic participants. **Methods:** Data from three prospective clinical studies evaluating the mannan-conjugated birch pollen allergoid EP-088-T502—T502-SIT-020, T502-SIT-045, and T502-SIT-068—were assembled for this cross-study comparison. Placebo-injection cohorts from studies T502-SIT-020 and T502-SIT-045 were compared with the injection-free cohort from study T502-SIT-068. Clinical outcomes included the Combined Symptom and Medication Score (CSMS), daily Symptom Score (dSS), and daily Medication Score (dMS). **Results:** The primary peak-period analysis included 60, 93, and 160 participants from T502-SIT-020, T502-SIT-045, and T502-SIT-068, respectively. During the site-specific 14-day peak birch pollen periods, median CSMS was 2.14 in the injection-free cohort compared with 1.34 and 1.07 in the T502-SIT-020 and T502-SIT-045 placebo-injection cohorts, respectively. These values corresponded descriptively to approximately 37% and 50% lower cohort medians, respectively, relative to the injection-free cohort. Median dSS, which was defined consistently across the three source studies, was 1.22 in the injection-free cohort compared with 0.78 and 0.70 in the two placebo-injection cohorts. Median dMS was 0.93 compared with 0.39 and 0.36, respectively. The same between-cohort pattern was observed in the secondary fixed 1–30 April analysis. **Conclusions:** Both placebo-injection cohorts showed lower CSMS and dSS values than the injection-free cohort. Because the cohorts were non-concurrent and derived from separate studies, the observed differences do not establish a causal effect of placebo injections.

## 1. Introduction

Randomised, double-blind, placebo-controlled (DBPC) trials are considered the reference standard for evaluating treatment efficacy because they allow specific treatment effects to be distinguished from nonspecific improvements that occur independently of the active intervention. Within this framework, placebo groups are essential for accounting for seasonal variations in pollen exposure, the natural course of allergic disease, and psychobiological effects associated with participation in a clinical trial [1].

The placebo effect refers to the improvement of symptoms following an intervention perceived as beneficial despite the absence of an active therapeutic component. By contrast, the placebo response refers to the overall change observed in placebo-treated participants and may also include natural disease variation, regression to the mean, environmental influences, and other nonspecific factors [1]. It reflects the influence of conditioning, patient expectations, and the therapeutic environment on both physiological responses and patients’ symptom perception. These effects are mediated by complex neurobiological mechanisms involving reward- and anxiety-related brain networks as well as bidirectional interactions between the nervous and immune systems [2].

Placebo responses in allergen immunotherapy (AIT) trials are frequently substantial and clinically relevant. Placebo-treated patients commonly experience improvements in allergic symptoms and reduced use of anti-allergic medication during the pollen season. For example, an analysis of six DBPC studies of sublingual and subcutaneous allergen immunotherapy (SLIT and SCIT), including birch pollen AIT studies, demonstrated reductions of up to 41% in the Combined Symptom and Medication Score (CSMS) among placebo-treated patients receiving SCIT [3]. However, improvement observed within a placebo group should not, by itself, be interpreted as a pure placebo effect, as natural disease variation and other nonspecific influences may also contribute [1].

The European Academy of Allergy and Clinical Immunology (EAACI) position paper reported improvements of up to 77% relative to pretreatment scores in placebo-treated groups in controlled AIT trials [1]. Factors influencing placebo responses include treatment frequency and invasiveness, patients’ expectations, previous treatment experiences, and the integrity of study blinding [2,4]. Regular clinical visits, repeated injections, and close medical monitoring may further reinforce patients’ perception of receiving effective therapy.

Despite the importance of placebo effects in clinical trial methodology, the clinical burden of birch pollen allergy remains considerable. Birch pollen is among the most important causes of seasonal allergic rhinitis (AR) in Central and Northern Europe [5]. Among tree pollens, birch (*Betula*) is the most allergenic species, followed by alder (*Alnus*) and hazel (*Corylus*). The prevalence of birch pollen allergy varies considerably across geographical regions, with positive skin prick tests reported in 5% to 54% of patients [6]. In most individuals, birch pollen allergy manifests as intermittent allergic rhinoconjunctivitis (ARC), although persistent ARC and asthma may also occur [7,8].

The management of pollen-induced ARC primarily relies on symptomatic pharmacotherapy. However, these therapies often fail to provide adequate symptom control in patients with moderate-to-severe disease [9] for whom AIT is recommended [10]. AIT is based on the repeated administration of an allergen, typically over a period of three to five years, with the aim of inducing long-term immune tolerance. It is one of the few disease-modifying treatment options for ARC. Numerous studies have demonstrated that AIT reduces allergic symptoms, decreases the use of anti-allergic medication, and provides sustained clinical benefits after treatment has been completed [10].

To further optimise AIT, a mannan-conjugated birch pollen allergoid (EP-088-T502) was developed for subcutaneous administration. Its efficacy and safety were evaluated in several clinical studies, including the dose-finding study T502-SIT-020 and the pivotal phase III DBPC study T502-SIT-045 [11,12]. These studies also included large, well-characterised placebo groups that demonstrated clinically relevant symptom improvement despite the absence of active AIT.

The present exploratory post hoc analysis compared allergic symptoms and rescue-medication use in placebo-injection cohorts from studies T502-SIT-020 and T502-SIT-045 with those in an injection-free cohort from study T502-SIT-068, in which the planned pre-seasonal injections were not administered before the 2024 birch pollen season. As the CSMS is a commonly used composite outcome in AIT trials [4], this comparison was intended to provide descriptive information on differences in patient-reported clinical outcomes between cohorts exposed to different study contexts. Because the cohorts were enrolled in separate studies conducted in different years and were not randomised for the comparison evaluated here, the analysis was not designed to estimate a causal effect of placebo injections.

## 2. Materials and Methods

### 2.1. Study Design

This post hoc analysis was based on data from three clinical studies (T502-SIT-020, T502-SIT-045, and T502-SIT-068). The source studies were conducted during the following periods: T502-SIT-020 from November 2019 to May 2020, T502-SIT-045 from November 2021 to June 2022, and T502-SIT-068 from 4 March 2024 to 14 June 2024.

All three studies were originally designed as DBPC trials to evaluate the efficacy and safety of SCIT with the mannan-conjugated birch pollen allergoid EP-088-T502. The three source studies shared several core eligibility criteria and outcome assessments but differed in calendar year, treatment implementation, visit schedule, and other study procedures. These characteristics are summarised in Table 1.

In studies T502-SIT-020 and T502-SIT-045, participants were randomised to receive either active treatment or placebo. Participants assigned to the active treatment group received EP-088-T502, whereas placebo-treated participants received saline solution according to the study injection schedule. All placebo-treated participants underwent regular clinical follow-up in accordance with the study protocol.

Study T502-SIT-068 received regulatory authorisation in Germany on 14 February 2024, and the first participant was enrolled on 4 March 2024. The planned treatment phase comprised five pre-seasonal treatment visits (V2–V6) over 5–12 weeks. Because the resulting late study start did not allow the planned pre-seasonal treatment schedule to be completed before the unusually early onset of the 2024 birch pollen season, the investigators decided for safety reasons not to administer study treatment and omitted visits V2–V6. The omission of treatment visits V2–V6 was recorded as a major protocol deviation. Consequently, neither active treatment nor placebo was administered, and the prepared randomisation list was not used. Because the treatment visits V2–V6 were omitted and no study injections were administered, participants in T502-SIT-068 could not remain blinded with respect to the absence of injections. They nevertheless underwent protocol-defined clinical assessments, including daily documentation of allergic symptoms and rescue-medication use as well as quality-of-life assessments. For the present analysis, participants with evaluable data formed an injection-free comparison cohort and were compared with the placebo-injection cohorts from studies T502-SIT-020 and T502-SIT-045.

### 2.2. Study Population

Eligible participants were adults aged 18–64 years with a documented history of moderate-to-severe AR or ARC caused by birch pollen during at least the two preceding pollen seasons, with disease severity classified according to ARIA. Additional eligibility criteria included a positive skin prick test to birch pollen and birch pollen-specific IgE of CAP class 3 or higher. In study T502-SIT-068, participants with CAP class 2 could also be included if a previous laboratory report, obtained outside the birch pollen season and no more than two years earlier, documented CAP class 3 or higher.

In study T502-SIT-020, 61 participants were randomised to the placebo group. In study T502-SIT-045, 99 participants received placebo treatment. In study T502-SIT-068, 162 eligible participants received no study injections; 161 completed the study and formed the injection-free comparison cohort. An overview of patient enrolment and treatment allocation across all three studies is provided in Table S1.

### 2.3. Combined Symptom and Medication Score

The CSMS, previously described in earlier studies, was selected as the primary endpoint of the present analysis [4,11,12].

#### 2.3.1. Daily Symptom Score (dSS)

Participants recorded nasal symptoms (rhinorrhoea, sneezing, nasal pruritus, and nasal congestion) and ocular symptoms (ocular pruritus and watery eyes) daily throughout the birch pollen season using the CCC STUDY Diary mobile application. Each symptom was assessed on a 4-point ordinal scale.

The daily Symptom Score (dSS) was calculated as the mean score of the six individual symptoms:

dSS = (SS1 + SS2 + SS3 + SS4 + SS5 + SS6)/6

Symptom severity was defined as follows:

**Score 0:** no symptoms

**Score 1:** mild symptoms (symptoms clearly present but easily tolerated)

**Score 2:** moderate symptoms (symptoms causing noticeable discomfort but remaining tolerable)

**Score 3:** severe symptoms (symptoms difficult to tolerate and interfering with daily activities and/or sleep)

#### 2.3.2. Daily Medication Score (dMS)

The CSMS methodology was based on the EAACI standardisation framework [4] and the approaches reported in the source studies [11,12]. For the present cross-study analysis, the daily Medication Score (dMS) was calculated using the adapted additive scoring approach described by Mösges et al. [12] for all three cohorts, including T502-SIT-020. This approach was developed based on experience from the earlier study [11], in which oral corticosteroid use was uncommon. Rescue medications could be used in any order or combination and were scored as follows: 0 = no medication; 0.5 = antihistamine eye drops; 1.0 = antihistamine tablets (desloratadine 5 mg once daily); and 1.5 = intranasal corticosteroid (fluticasone furoate nasal spray, 110 μg/day). The daily dMS was calculated as the sum of the medication scores used on that day. In all three cohorts, the daily CSMS was calculated as the sum of the dSS and dMS (CSMS = dSS + dMS).

### 2.4. Pollen Data

The site-specific peak birch pollen period was defined as the 14 consecutive days within 1–30 April with the highest predicted birch pollen levels. For the present cross-study comparison, regional DWD (Deutscher Wetterdienst, German Weather Forecast) forecast data were assembled for all three study years and used to derive a comparable descriptive pollen-exposure index across study sites. DWD forecast categories were converted to a numerical index ranging from 0 to 3 for descriptive summarisation. Mean values therefore represent averages of the forecast index across study centres and should not be interpreted as measured pollen concentrations or individual pollen exposure. For a secondary calendar-based analysis, the fixed period from 1 to 30 April was used in all three studies.

### 2.5. Rhinoconjunctivitis Quality of Life Questionnaire

Health-related quality of life (QoL) was assessed using the Rhinoconjunctivitis Quality of Life Questionnaire (RQLQ), originally developed by Juniper and Guyatt [13] and administered in its validated German version [14,15]. The questionnaire is specifically designed to assess disease-related impairment in patients with ARC. The RQLQ was completed at protocol-defined study visits before and during the birch pollen season in all three source studies. Because visit timing was study-specific and occurred in different calendar years, these assessments were not assumed to represent identical levels of seasonal pollen exposure across cohorts.

### 2.6. Statistical Analysis

Statistical analyses were performed using IBM SPSS Statistics version 30 (IBM Corp., Armonk, NY, USA). Overall comparisons among the three cohorts were performed using the Kruskal–Wallis test. Pairwise comparisons were performed using two-sided Mann–Whitney U tests. The reported *p*-values are presented as provided in the cross-study statistical output and should be interpreted in the context of this exploratory post hoc analysis. For the present cross-study analysis, missing diary data were handled using multiple imputation. For T502-SIT-068, the source analysis specified a Missing at Random assumption, with dSS and dMS imputed before calculation of CSMS. A harmonised imputation and averaging procedure across all three source studies could not be confirmed from the available documentation.

The primary endpoint was the CSMS in the injection-free cohort from study T502-SIT-068 and the placebo-injection cohorts from studies T502-SIT-020 and T502-SIT-045. The site-specific 14-day peak birch pollen period was considered the primary analysis window, whereas the fixed 1–30 April period was used as a secondary supporting analysis. Secondary endpoints included the dSS, dMS, and QoL.

Descriptive statistics, including the number of participants, mean, standard deviation, median, quartiles, minimum, and maximum, were calculated for both the site-specific 14-day peak birch pollen period and the fixed 1–30 April analysis period. For studies T502-SIT-020 and T502-SIT-045 [11,12], analyses were performed using the intention-to-treat (ITT) population. For study T502-SIT-068, the Eligible Set (E-Set) comprised all participants who provided written informed consent and fulfilled the inclusion and exclusion criteria and who would have received study treatment according to the original protocol. Of the 162 participants in the E-Set, 161 completed the study and were included in the comparative outcome analyses. The analysis-specific Valid N values reported for the 14-day peak analysis were 60, 93, and 160 for T502-SIT-020, T502-SIT-045, and T502-SIT-068, respectively; for the fixed 1–30 April analysis, the corresponding values were 60, 93, and 161. The available documentation did not specify the reason for each difference between the source-cohort size and the analysis-specific Valid N. All between-study comparisons were exploratory and were not intended to estimate a causal effect of placebo injections.

### 2.7. Ethical and Regulatory Considerations

Study T502-SIT-068 was conducted in accordance with the Declaration of Helsinki (Fortaleza 2013) [16], the International Council for Harmonisation Good Clinical Practice guidelines (ICH-GCP; CPMP/ICH/135/95), and applicable national drug, data protection, and other regulatory requirements.

The participant information sheet, informed consent form, and data protection declaration were approved by the responsible ethics committee before participant enrolment. The available study documentation confirms the original ethics-approved informed-consent process. The documentation available for the present analysis did not provide information on whether a separate ethics-committee notification, protocol amendment, or re-consent procedure was undertaken specifically in relation to the subsequent omission of treatment visits V2–V6, or whether participants received separate written information regarding continuation in the study without study injections. The study was submitted through the Clinical Trials Information System (CTIS) and received regulatory authorisation in Germany from the Paul-Ehrlich-Institut on 14 February 2024. Trial registration identifiers were EudraCT 2018-002522-23 for T502-SIT-020, EudraCT 2021-002252-36 for T502-SIT-045, and EU CT 2023-508013-16-00 for T502-SIT-068

## 3. Results

### 3.1. Study and Cohort Characteristics

Of 236 participants screened for study T502-SIT-068, 74 were not enrolled, leaving 162 eligible participants in the E-Set. One participant discontinued because of an adverse event (AE). No serious or other significant AEs occurred during the study. Therefore, 161 participants completed the study and were included in the comparative outcome analyses. Further details are provided in Figure S1. Key study and cohort characteristics relevant to the present cross-study comparison are summarised in Table 1.

Selected baseline and study-procedure characteristics of the three source cohorts are summarised in Table 1, with additional baseline characteristics provided in Table S2. The cohorts were similar in age, sex distribution, and asthma prevalence among the selected characteristics shown, but differed in other clinical and procedural features, including pre-season RQLQ values and treatment-visit structure. For the present cross-study analysis, the same adapted additive dMS scoring approach was applied across all three cohorts. Mean age ranged from 37 to 40 years, the proportion of female participants from 45.9% to 52.5%, and asthma prevalence from 25.3% to 30.9%. Full baseline characteristics for studies T502-SIT-020 and T502-SIT-045 have been reported previously [11,12]. Mean regional DWD peak pollen forecast levels on the 0–3 index were 2.99 in 2020, 2.85 in 2022, and 2.25 in 2024 (Table S3).

Daily symptom and rescue-medication data were recorded electronically in all three source studies. The placebo-injection cohorts underwent regular protocol-defined clinical follow-up, whereas in T502-SIT-068 the planned treatment visits V2–V6 were omitted while protocol-defined clinical assessments continued. Comparable diary-completion rates and harmonised measures of investigator contact across the three cohorts were not available for the present cross-study comparison.

All included participants had a history of birch pollen-induced AR or ARC, with or without asthma, for at least 2 years and had used anti-allergic medication during at least 2 previous birch pollen seasons.

### 3.2. Between-Cohort Differences in Combined Symptom and Medication Score (CSMS)

During the site-specific 14-day peak birch pollen period, defined as the 14 consecutive days in April with the highest predicted birch pollen levels, participants who received no study injections in study T502-SIT-068 experienced a significantly greater burden of allergic symptoms and rescue-medication use than participants in the placebo groups of studies T502-SIT-020 and T502-SIT-045. Specifically, participants in the injection-free cohort reported more severe symptoms and required more rescue medication than participants who received placebo injections.

The median CSMS during the peak birch pollen season was 1.34 in study T502-SIT-020, 1.07 in study T502-SIT-045, and 2.14 in study T502-SIT-068. Pairwise Mann–Whitney U comparisons showed significant between-cohort differences between T502-SIT-068 and each placebo-injection cohort (*p* < 0.001 for both comparisons). Descriptively, the median CSMS in T502-SIT-068 was 1.6-fold higher than in T502-SIT-020 and 2.0-fold higher than in T502-SIT-045 (Figure 1A).

During the fixed 1–30 April analysis period, the median CSMS was 1.32 in study T502-SIT-020, 0.86 in study T502-SIT-045, and 1.97 in study T502-SIT-068. Pairwise Mann–Whitney U comparisons showed significant between-cohort differences between T502-SIT-068 and each placebo-injection cohort (*p* < 0.001 for both comparisons). Descriptively, the median CSMS in T502-SIT-068 was 1.5-fold higher than in T502-SIT-020 and 2.3-fold higher than in T502-SIT-045 (Figure 1B).

### 3.3. Between-Cohort Differences in Daily Symptom Score (dSS)

During the peak birch pollen season, the median dSS was 0.78 in study T502-SIT-020, 0.70 in study T502-SIT-045, and 1.22 in study T502-SIT-068. Pairwise Mann–Whitney U comparisons showed significant between-cohort differences between T502-SIT-068 and each placebo-injection cohort (*p* < 0.001 for both comparisons). Descriptively, the median dSS in T502-SIT-068 was 1.6-fold higher than in T502-SIT-020 and 1.7-fold higher than in T502-SIT-045 (Figure 2A).

Analysis of the fixed 1–30 April period showed that symptom scores were higher in the injection-free cohort. The median dSS was 0.79 in study T502-SIT-020, 0.59 in study T502-SIT-045, and 1.08 in study T502-SIT-068. Pairwise Mann–Whitney U comparisons showed significant between-cohort differences between T502-SIT-068 and each placebo-injection cohort (*p* < 0.001 for both comparisons). Descriptively, the median dSS in T502-SIT-068 was 1.4-fold higher than in T502-SIT-020 and 1.8-fold higher than in T502-SIT-045 (Figure 2B).

### 3.4. Between-Cohort Differences in Daily Medication Score (dMS)

During the peak birch pollen season, the median dMS was 0.39 in study T502-SIT-020, 0.36 in study T502-SIT-045, and 0.93 in study T502-SIT-068. Pairwise Mann–Whitney U comparisons showed significant between-cohort differences between T502-SIT-068 and each placebo-injection cohort (*p* < 0.001 for both comparisons). Descriptively, the median dMS in T502-SIT-068 was 2.4-fold higher than in T502-SIT-020 and 2.6-fold higher than in T502-SIT-045 (Figure 3A).

Consistent with these findings, rescue medication use remained higher in the injection-free cohort during the fixed 1–30 April analysis period. The median dMS was 0.33 in study T502-SIT-020, 0.25 in study T502-SIT-045, and 0.87 in study T502-SIT-068. Pairwise Mann–Whitney U comparisons showed significant between-cohort differences between T502-SIT-068 and each placebo-injection cohort (*p* < 0.001 for both comparisons). Descriptively, the median dMS in T502-SIT-068 was 2.6-fold higher than in T502-SIT-020 and 3.5-fold higher than in T502-SIT-045 (Figure 3B).

### 3.5. Between-Cohort Comparison of RQLQ Scores

In addition to the CSMS, health-related quality of life was assessed using the RQLQ across all three studies. Before the onset of the birch pollen season, mean RQLQ scores were 0.77 in study T502-SIT-020, 1.41 in study T502-SIT-045, and 1.36 in the injection-free cohort of study T502-SIT-068 (Figure 4A).

During the birch pollen season, mean RQLQ scores were 1.61 in study T502-SIT-020, 1.49 in study T502-SIT-045, and 1.72 in the injection-free cohort of study T502-SIT-068 (Figure 4B).

RQLQ scores are presented descriptively, and no clear between-cohort pattern was observed during the birch pollen season. A harmonised paired within-participant change analysis was not available across all three source cohorts; therefore, differences between the visit-level group means were not interpreted as within-participant changes.

## 4. Discussion

In this exploratory post hoc analysis, both placebo-injection cohorts showed lower CSMS, dSS, and dMS values than the injection-free cohort. During the primary site-specific 14-day peak period, median CSMS was 2.14 in T502-SIT-068 compared with 1.34 in T502-SIT-020 and 1.07 in T502-SIT-045, corresponding descriptively to approximately 37% and 50% lower cohort medians in the two placebo-injection cohorts. These percentages represent descriptive relative differences between cohort medians, calculated using the injection-free cohort median as the reference. Importantly, the consistently defined dSS showed the same direction of between-cohort differences, with median values of 1.22 in T502-SIT-068 compared with 0.78 and 0.70 in T502-SIT-020 and T502-SIT-045, respectively. The CSMS and dSS findings showed a consistent between-cohort pattern. Because the cohorts were derived from separate, non-concurrent studies and were not randomised for the present comparison, this pattern cannot be attributed specifically to placebo injections.

The CSMS is a recommended composite outcome for clinical trials in AIT [4] and has been used as an efficacy endpoint in the source studies [11,12].

Previous analyses of SCIT and SLIT trials have reported reductions in CSMS of up to 40.5% within placebo groups, depending on study design and patient expectations [3,17]. The EAACI position paper reported improvements of up to 77% relative to pretreatment scores in placebo-treated groups in controlled AIT trials [1]. This response is influenced by several factors, including treatment frequency, increased medical attention, patient expectations, previous treatment experience, and the integrity of study blinding [1,2,4]. The findings of the present post hoc analysis highlight the potential relevance of the therapeutic context, including regular physician visits, repeated injections, and continuous patient monitoring, when interpreting patient-reported outcomes (PROs). The observed between-cohort differences may involve placebo-associated and other trial-related mechanisms; however, the present analysis was not designed to distinguish among these potential mechanisms. RQLQ results did not show the same between-cohort separation as the diary-based symptom and medication outcomes. Moreover, pre-season RQLQ values differed across the three source cohorts, which limits direct comparison of the subsequent in-season values. The timing of the RQLQ assessments relative to seasonal pollen exposure was also not fully comparable across studies. In T502-SIT-045, the source publication notes that the V7 assessment may already have been influenced by substantial pollen exposure in March 2022. In T502-SIT-068, V7 was scheduled before and V8 at the peak of the birch pollen season, although deviations from the planned visit timing occurred in a small number of participants. These differences in baseline values and assessment timing further limit cross-study interpretation of the RQLQ findings.

Doctor–patient interactions, patient expectations, and regular follow-up visits are recognised contextual factors that may influence symptom perception and the treatment experience. In the present analysis, lower symptom scores were observed in the placebo-injection cohorts than in the injection-free cohort; however, expectations, therapeutic interactions, and adherence-related mechanisms were not directly assessed. Treatment adherence remains an important consideration in AIT, which generally requires prolonged treatment to achieve sustained clinical benefit [10,18,19]. Broader placebo literature has described neurobiological mechanisms that may contribute to placebo responses [20,21]; however, such mechanisms were not assessed in the present study. Communication, patient education, and structured follow-up have also been discussed as factors relevant to treatment persistence in the AIT literature [18,19,22]. These considerations provide broader clinical context but cannot explain the between-cohort differences observed in the present analysis.

Furthermore, the inclusion of an injection-free comparison cohort may provide additional insights in future AIT studies. Most AIT trials compare active treatment with placebo alone and therefore do not evaluate the contribution of placebo responses relative to an injection-free trial context. A concurrent injection-free comparison arm could be included prospectively; however, participants could not be blinded to whether they received study injections, which would need to be considered when interpreting expectation- and context-related effects. The present comparison provides exploratory information on differences between placebo-injection and injection-free trial contexts but does not permit the observed between-cohort differences to be interpreted as a quantified placebo response or placebo effect. These findings may help optimise the design and interpretation of future AIT trials, in which careful evaluation of placebo responses is essential to avoid underestimating treatment efficacy.

### Limitations

This study has several limitations. First, the comparison was non-randomised and non-concurrent, as the cohorts were derived from three separate studies conducted in 2020, 2022, and 2024. Differences in pollen exposure, participant characteristics, study procedures, blinding, visit schedules, expectations, natural between-season variation, reporting effects, and other unmeasured factors may therefore have contributed to the observed between-cohort differences. These between-study differences could not be fully adjusted post hoc. A harmonised imputation and averaging procedure across all three source studies could not be confirmed from the available documentation, and differences in other source-study procedures remain a limitation of the cross-study comparison. The fixed 1–30 April analysis window may have captured different proportions of the actual birch pollen season in the three study years, introducing additional temporal and exposure-related confounding. In addition, the present analysis used regional DWD forecast data rather than harmonised site- and date-matched measured pollen counts. Individual pollen exposure could therefore not be quantified, and the reported forecast averages should not be interpreted as demonstrating equivalent exposure across cohorts. No analysis restricted to overlapping study sites or regions was performed. Potential co-exposure to other seasonal pollens, including alder and hazel, was not assessed in the present cross-study analysis.

In addition, study T502-SIT-020 was conducted during the first COVID-19 lockdown in Germany, when reduced outdoor activity, face-mask use, and other behavioural changes may have altered allergen exposure and symptom reporting, providing an alternative explanation for the lower scores observed in this cohort. The analysis cannot isolate the effect of placebo injections from these cross-study differences and should be regarded as exploratory and hypothesis-generating.

Mechanistic and immunological biomarkers may provide complementary information when investigating biological responses to AIT [23]. However, the present cross-study analysis was based primarily on patient-reported symptom and rescue-medication outcomes, and harmonised mechanistic measures were not available across the three source studies. Such biomarkers could be informative in future prospective studies but cannot retrospectively determine the cause of the between-cohort differences observed here. Therefore, the mechanisms underlying the observed between-cohort differences could not be assessed.

## 5. Conclusions

In this exploratory post hoc cross-study comparison, both placebo-injection cohorts showed lower symptom and combined symptom–medication scores than the injection-free cohort. During the primary 14-day peak period, median CSMS was descriptively approximately 37% lower in T502-SIT-020 and 50% lower in T502-SIT-045 than in T502-SIT-068, while the consistently defined dSS showed the same direction of between-cohort differences. Because the cohorts were derived from separate, non-concurrent studies, the observed differences should be interpreted descriptively and do not establish a causal effect of placebo injections. They nevertheless support further prospective investigation of injection-related and broader contextual influences on patient-reported outcomes in AIT.

## Figures and Tables

**Figure 1:**
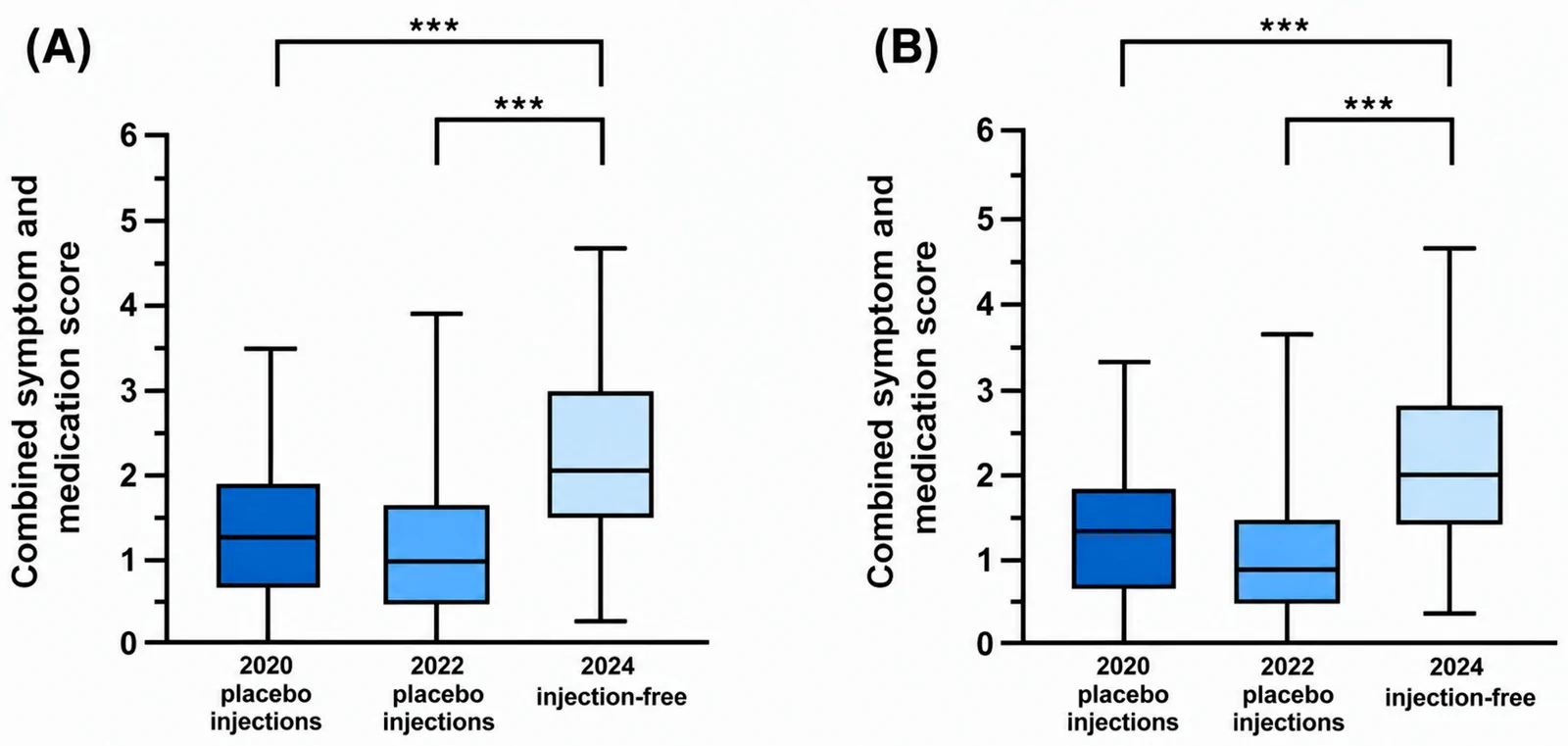
Combined Symptom and Medication Score (CSMS) in the 2020 placebo-injection cohort (T502-SIT-020), 2022 placebo-injection cohort (T502-SIT-045), and 2024 injection-free cohort (T502-SIT-068). (**A**) Site-specific 14-day peak birch pollen period. (**B**) Fixed 1–30 April analysis period. Data are presented as medians, 25th–75th percentiles, and minimum–maximum values. *** *p* < 0.001.

**Figure 2:**
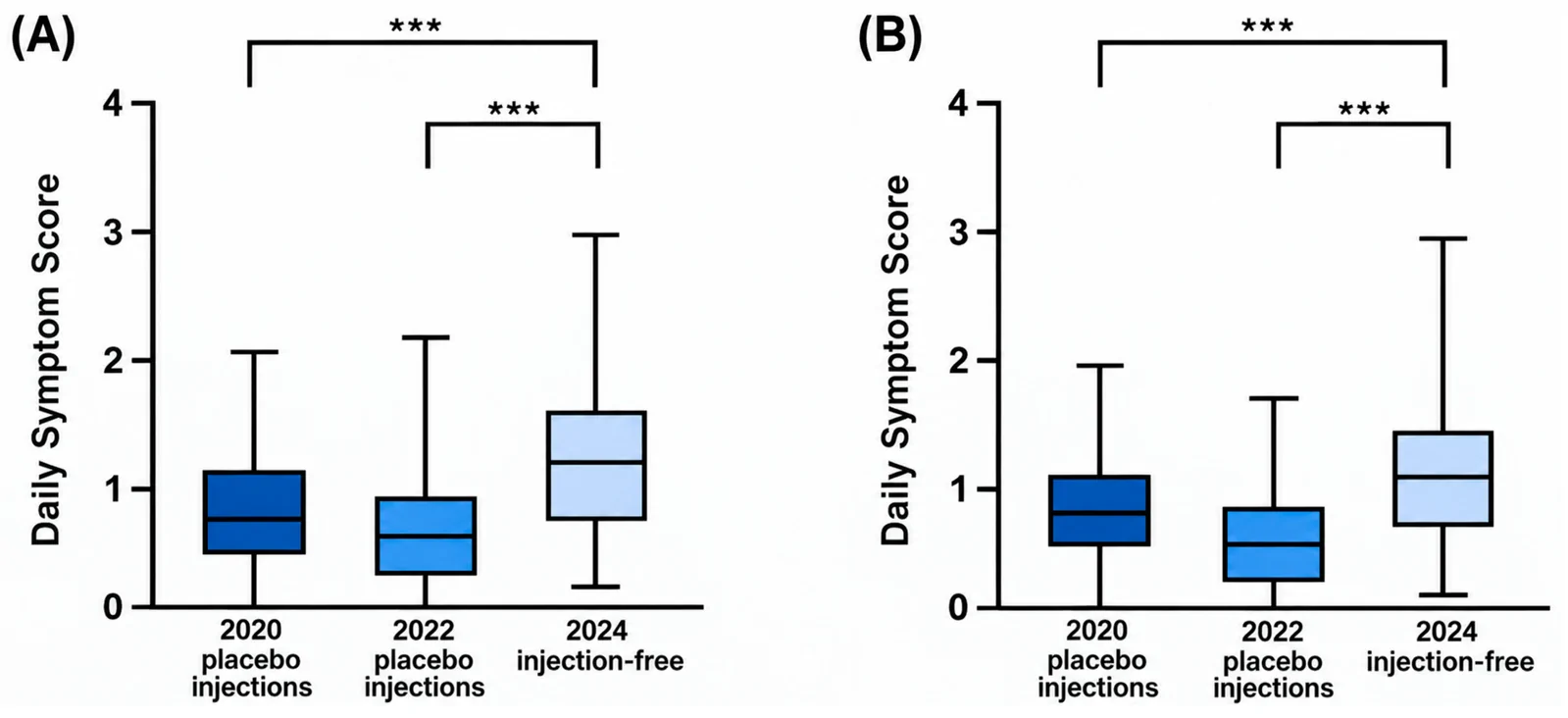
Daily Symptom Score (dSS) in the 2020 placebo-injection cohort (T502-SIT-020), 2022 placebo-injection cohort (T502-SIT-045), and 2024 injection-free cohort (T502-SIT-068). (**A**) Site-specific 14-day peak birch pollen period. (**B**) Fixed 1–30 April analysis period. Data are presented as medians, 25th–75th percentiles, and minimum–maximum values. *** *p* < 0.001.

**Figure 3:**
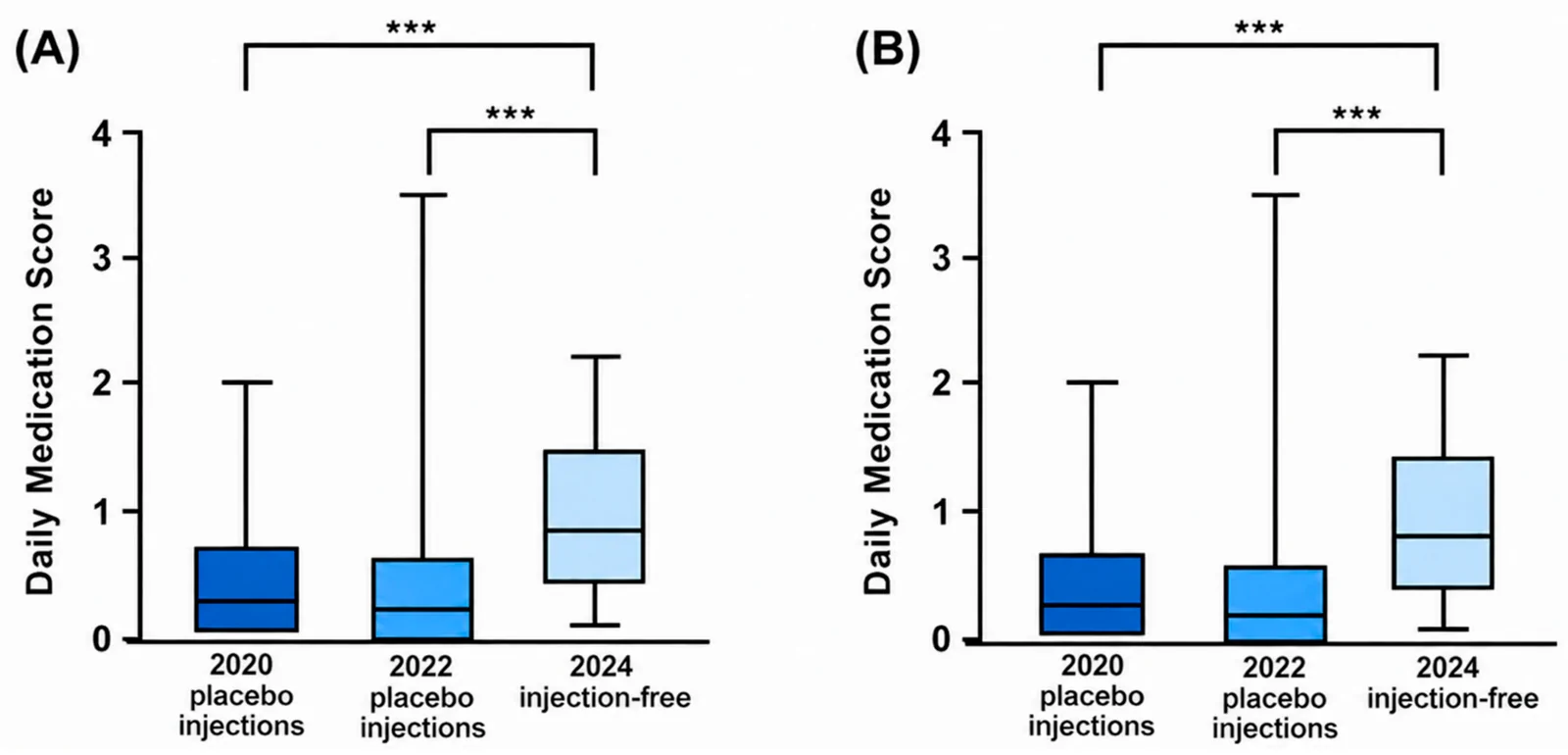
Daily Medication Score (dMS) in the 2020 placebo-injection cohort (T502-SIT-020), 2022 placebo-injection cohort (T502-SIT-045), and 2024 injection-free cohort (T502-SIT-068). (**A**) Site-specific 14-day peak birch pollen period. (**B**) Fixed 1–30 April analysis period. Data are presented as medians, 25th–75th percentiles, and minimum–maximum values. *** *p* < 0.001.

**Figure 4:**
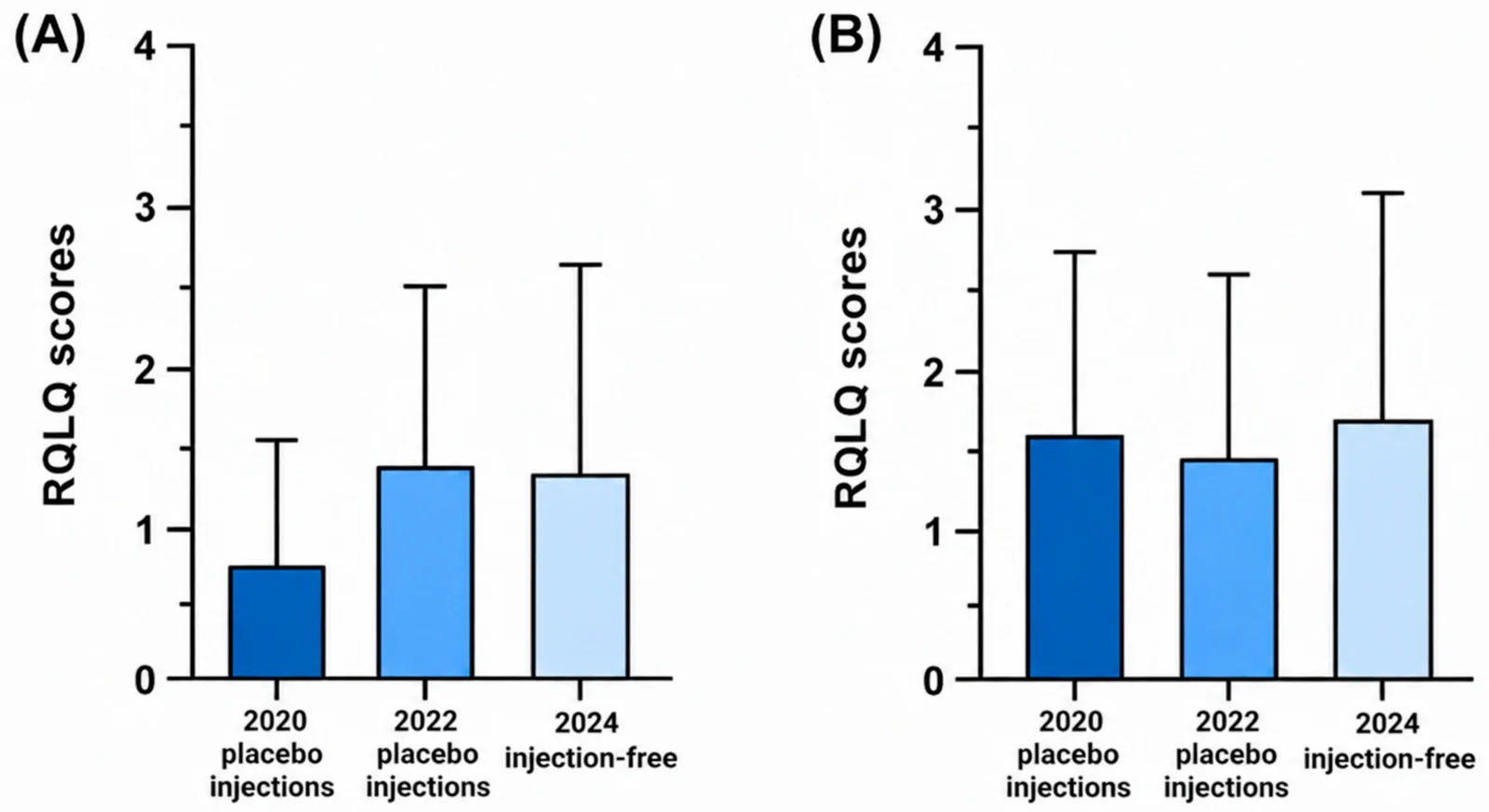
Rhinoconjunctivitis Quality of Life Questionnaire (RQLQ) scores in the 2020 placebo-injection cohort (T502-SIT-020), 2022 placebo-injection cohort (T502-SIT-045), and 2024 injection-free cohort (T502-SIT-068). (**A**) RQLQ scores immediately before the birch pollen season. (**B**) RQLQ scores during the birch pollen season. Data are presented as mean ± SD.

**Table 1:** Key study and cohort characteristics relevant to the present cross-study comparison.

Characteristic	T502-SIT-020	T502-SIT-045	T502-SIT-068
**Study period**	Nov 2019–May 2020	Nov 2021–Jun 2022	Mar–Jun 2024
**Study sites**	21 sites, Germany	28 sites, Germany	20 sites planned in Germany; Polish sites did not enrol participants
**Original study design**	Phase IIa/IIb randomised, double-blind, placebo-controlled dose-finding trial	Phase III randomised, double-blind, placebo-controlled trial	Phase III randomised, double-blind, placebo-controlled trial as planned; treatment phase not conducted
**Cohort included in present comparison**	Placebo-injection cohort	Placebo-injection cohort	Injection-free cohort
**Baseline cohort, *n***	61	99	162
**Valid N, 14-day peak analysis**	60	93	160
**Valid N, 1–30 April analysis**	60	93	161
**Age, years, mean ± SD**	37.7 ± 10.0	37 ± 13	40 ± 11
**Female, *n* (%)**	28 (45.9)	50 (50.5)	85 (52.5)
**Asthma, *n* (%)**	17 (27.9)	25 (25.3)	50 (30.9)
**Pre-season RQLQ, mean**	0.77	1.41	1.36
**Core allergy criteria**	AR/ARC ≥ 2 seasons; positive birch SPT; birch-specific IgE CAP ≥ 3; anti-allergic medication ≥ 2 seasons;	AR/ARC ≥ 2 seasons; positive birch SPT; birch-specific IgE CAP ≥ 3; anti-allergic medication ≥ 2 seasons	AR/ARC ≥ 2 seasons; positive birch SPT; birch-specific IgE CAP ≥ 3, with protocol-defined allowance for CAP 2 if previous off-season CAP ≥ 3 was documented; anti-allergic medication ≥ 2 seasons
**Treatment-visit structure**	5 pre-seasonal treatment visits (V2–V6)	5 pre-seasonal treatment visits (V2–V6)	5 treatment visits planned (V2–V6), all omitted
**Placebo injections received by cohort**	6 injections over 5 visits	7 injections over 5 visits	None
**dMS scoring used in the present cross-study analysis**	Adapted additive dMS (Mösges score)	Adapted additive dMS (Mösges score)	Adapted additive dMS (Mösges score)

## Data Availability

Data will be available from the corresponding author upon reasonable request.

## Supplementary Materials

The following supporting information can be downloaded at: https://www.mdpi.com/article/10.3390/jcm15197637/s1, Figure S1: Flow chart of the study. Of note, no participants from Poland were screened or included; Table S1: Overview of patient enrolment and treatment allocation across trials T502-SIT-020, T502-SIT-045 and T502-SIT-068; Table S2: Baseline characteristics of the source cohorts; Table S3. Mean regional DWD birch pollen forecast levels during the site-specific peak birch pollen seasons in 2020, 2022, and 2024.

## Abbreviations

The following abbreviations are used in this manuscript:

**Abbreviation**	**Definition**
AE	Adverse event
AIT	Allergen immunotherapy
AR	Allergic rhinitis
ARIA	Allergic Rhinitis and its Impact on Asthma
ARC	Allergic rhinoconjunctivitis
CSMS	Combined Symptom and Medication Score
CTIS	Clinical Trials Information System
DBPC	Double-blind, placebo-controlled
dMS	Daily Medication Score
dSS	Daily Symptom Score
DWD	Deutscher Wetterdienst
EAACI	European Academy of Allergy and Clinical Immunology
E-Set	Eligible Set
ICH-GCP	International Council for Harmonisation Good Clinical Practice
IgE	Immunoglobulin E
ITT	Intention-to-treat
NPT	Nasal provocation test
PRO	Patient-reported outcome
QoL	Quality of Life
RQLQ	Rhinoconjunctivitis Quality of Life Questionnaire
SCIT	Subcutaneous allergen-specific immunotherapy
SD	Standard deviation
SLIT	Sublingual immunotherapy
SPT	Skin prick test
