# Supplementary material for: Clinical Outcomes in Placebo-Injection and Injection-Free Cohorts in Birch Pollen Allergy: An Exploratory Cross-Study Comparison
Source: J Clin Med. 2026 Oct 2;15(19):7637. doi: 10.3390/jcm15197637 (PMC13666366; doi:10.3390/jcm15197637)
Supplement: Supplementary file 1 [file jcm-15-07637-s001.zip › jcm-4513235-supplementary.pdf]

Supplementary Materials: The following supporting information can be downloaded at: https://www.mdpi.com... Figure S1: Flow chart of the study. Of note, no participants from Poland were screened or included; Table S1: Overview of patient enrolment and treatment allocation across trials T502-SIT-020, T502-SIT-045 and T502-SIT-068; Table S2: Baseline characteristics of the source cohorts; Table S3. Mean regional DWD birch pollen forecast levels during the site-specific peak birch pollen seasons in 2020, 2022, and 2024.

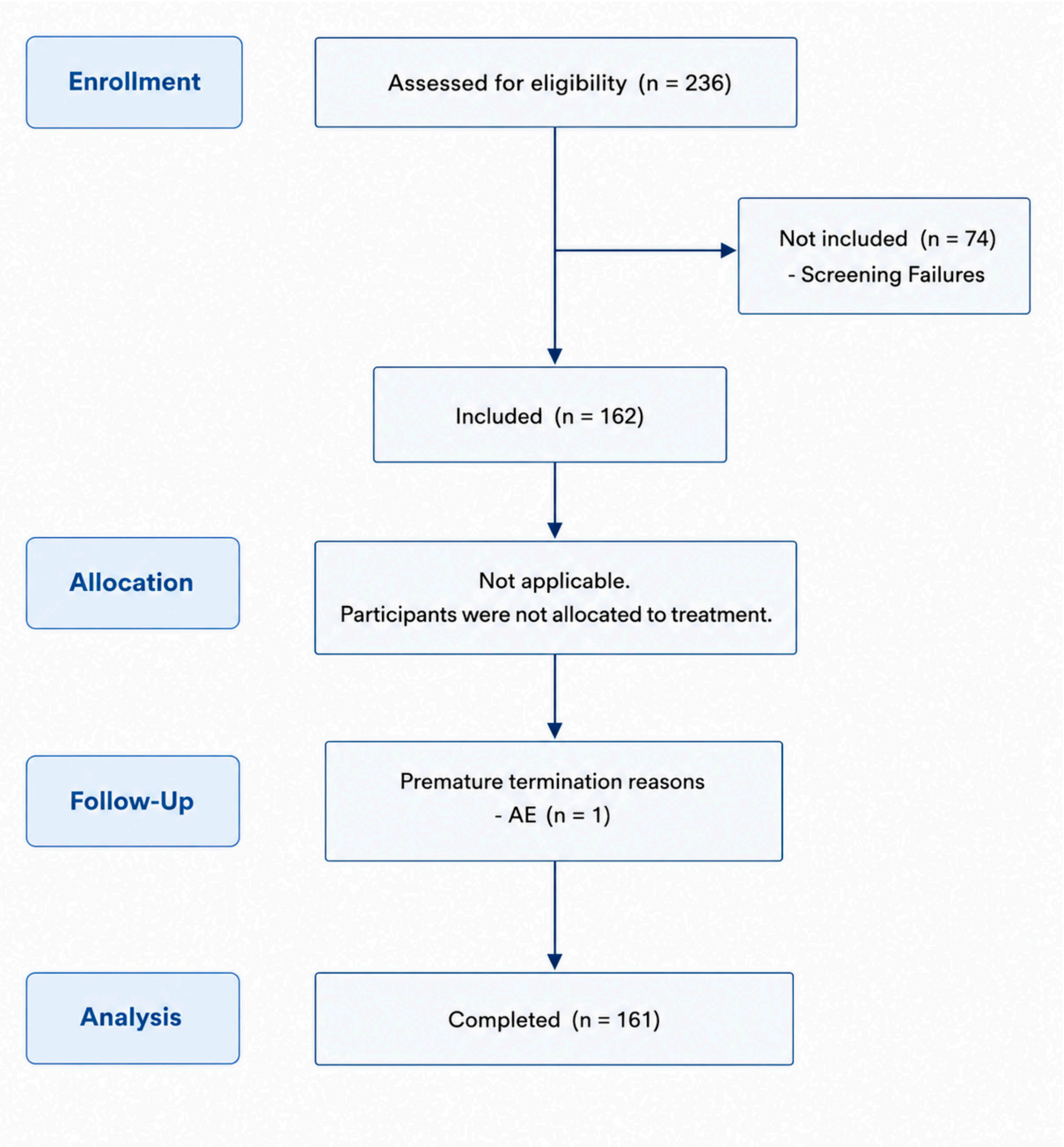

Figure S1: Flow chart of the study. Of note, no participants from Poland were screened or included.

Table S1: Overview of patient enrolment and treatment allocation across trials T502-SIT-020, T502-SIT-045 and T502-SIT-068.

Trial	Participants screened (n)	Participants randomised/completed (n)	Placebo Group (n)	Active Treatment Group (n)	Participants in the injection-free cohort (n)
T502-SIT-020	313	246	61	185	0
T502-SIT-045	405	298	99	199	0
T502-SIT-068	236	161	0	0	161
**Total**	**954**	**705**	**160**	**384**	**161**

Note: For studies T502-SIT-020 and T502-SIT-045, values represent randomised participants; for study T502-SIT-068, the value represents participants who completed the study.

Table S2: Baseline characteristics of the source cohorts

Characteristics	T502-SIT-020	T502-SIT-045	T502-SIT-068
Participants (n / %)	61 / 100%	99 / 100%	162 / 100%
Female (n / %)	28 / 45.9%	50 / 50.5%	85 / 52.5%
Male (n / %)	33 / 54.1%	49 / 49.5%	77 / 47.5%
Age in years (mean / median)	38 / 36	37 / 34	40 / 39
Asthma (n / %)	17 / 27.9%	25 / 25.3%	50 / 30.9%
Allergic asthma (n / %)	16 / 26.2%	24 / 24.2%	43 / 26.5%

Note: Baseline characteristics for T502-SIT-020 and T502-SIT-045 are based on the randomised placebo cohorts. Baseline characteristics for T502-SIT-068 are based on the E-Set of 162 eligible participants. One participant in T502-SIT-068 discontinued because of an AE, leaving 161 participants in the comparative outcome analyses.

Table S3. Mean regional DWD birch pollen forecast levels during the site-specific peak birch pollen seasons in 2020, 2022, and 2024.

Study	Year	Mean DWD peak pollen forecast	SD	Centres
T502-SIT-020	2020	2.99	0.04	15
T502-SIT-045	2022	2.85	0.13	15
T502-SIT-068	2024	2.25	0.20	17

Note: DWD, German Weather Service (Deutscher Wetterdienst); SD, standard deviation. DWD forecast categories were converted to numerical scores ranging from 0 to 3. The site-specific peak birch pollen period was defined as the 14 consecutive days within 1–30 April of the respective study year with the highest predicted birch pollen levels.
